# New altitudinal record for white-lipped peccary *Tayassu pecari* (Link, 1795) in Ecuador, with notes about activity patterns and herd size

**DOI:** 10.3897/BDJ.7.e33275

**Published:** 2019-04-17

**Authors:** Javier Torres, María Mercedes Gavilánez

**Affiliations:** 1 Universidad Central del Ecuador - Facultad de Ciencias Biológicas, Quito, Ecuador Universidad Central del Ecuador - Facultad de Ciencias Biológicas Quito Ecuador

**Keywords:** *Tayassu pecari*, new altitudinal record, Ecuadorian Amazon, small herds

## Abstract

White-lipped peccaries (*Tayassu pecari*) represent a key element of trophic networks in tropical rainforest ecosystems by playing the dual role of consumer and prey. Despite their importance, pressures on their populations have increased during the last few decades due to hunting and deforestation across its distributional range. These pressures may have led the remaining populations to move into new territories and to change their migratory and gregarious behaviour. In this study, we used photographic records from camera traps to collect data on biogeography of white-lipped peccaries in order to answer some questions about the demography, distribution and population size of the species in Ecuador’s western Amazonia. We present new altitudinal records for the species (2,000 metres above sea level), along with some notes on herd size and activity patterns. This information is valuable for obtaining a better understanding of the species distribution and population status in order to achieve better conservation plans.

## Introduction

White-lipped peccaries are distributed from Mexico to Argentina, occupying a wide range of habitats, commonly living in large herds of 50–300 individuals (Taber et al. 2008, Altrichter et al. 2012). Population densities vary widely according to seasonal, environmental and habitat conditions, ranging from 0.43 ind/km^2^ to 13.7 ind/km^2^ (Keuroghlian et al. 2004, Reyna-Hurtado and Tanner 2007, Reyna-Hurtado et al. 2009, Desbiez et al. 2010, Altrichter et al. 2012).

Across its entire range, it is known to occur at elevations up to 1,900 metres (Keuroghlian et al. 2013), while in Ecuador it has only been recorded between 0–1,600 metres above sea level (Tirira 2017). It is distributed on the eastern and western slopes of the Andes, inhabiting wet, dry, tropical and subtropical forests, in herds ranging from 50 to 300 or more individuals (Kiltie and Terborgh 1983, Mayer and Wetzel 1987, Sowls 1997, Fragoso 2004, Tirira 2017), although there are also records of much smaller herds (less than 10 individuals) (Moreira-Ramírez et al. 2015).

Peccaries are extremely important in food webs, both as prey and consumers (Garla et al. 2001, Moreno et al. 2006, Galetti et al. 2015, Briceño-Méndez et al. 2017). As consumers, due to their gregarious behaviour and primarily frugivorous diet, white lipped peccaries are considered ecosystem engineers, playing a key role in structuring plant communities in tropical rainforests (Keuroghlian and Eaton 2009, Beck et al. 2010, Ringler et al. 2015). As prey, peccaries are one of the largest contributors to the secondary production (biomass) in tropical rainforests (Taber et al. 2008, Altrichter et al. 2012), as a main food source for predators, allowing for the maintenance of stability in these ecosystems (Aranda 1994, Taber et al. 2008).

Despite their importance, their populations are decreasing due to anthropogenic pressures, such as over-hunting and deforestation (Cullen et al. 2000, Fragoso 2004, de Azevedo and Conforti 2008, Taber et al. 2008, Reyna-Hurtado 2009, Reyna-Hurtado et al. 2009, de Freitas et al. 2010, Altrichter et al. 2012, Keuroghlian et al. 2013). According to IUCN, white-lipped peccaries are considered Vulnerable (VU) throughout their range (Keuroghlian et al. 2013), while in Ecuador - according to the Red Book of Mammals - western populations are considered Critically Endangered and eastern populations are Endangered (Tirira 2001, Tirira 2017). The greatest local threats for the species are illegal hunting, habitat loss and disease (Tirira 2017), while throughout its geographical range, its population decline has been attributed to continued, widespread deforestation and intense hunting pressure from humans (Fragoso 2004, Keuroghlian et al. 2013). This species is particularly vulnerable to human presence and habitat fragmentation because they require large extents of undisturbed forest with sufficient food availability to meet their biological requirements (Moreira-Ramírez et al. 2015).

One of the main problems, not only for white-lipped peccaries, but wildlife in general in Ecuador, is the lack of information regarding demography, distribution and population size (Taber et al. 2008). These data are fundamental to understanding threats affecting local populations and establishing appropriate conservation and management strategies for the species at local and regional levels. The present study aims to fill some of these information gaps, thus contributing to the conservation of white-lipped peccaries.

## Materials and Methods

**Figure 1.**
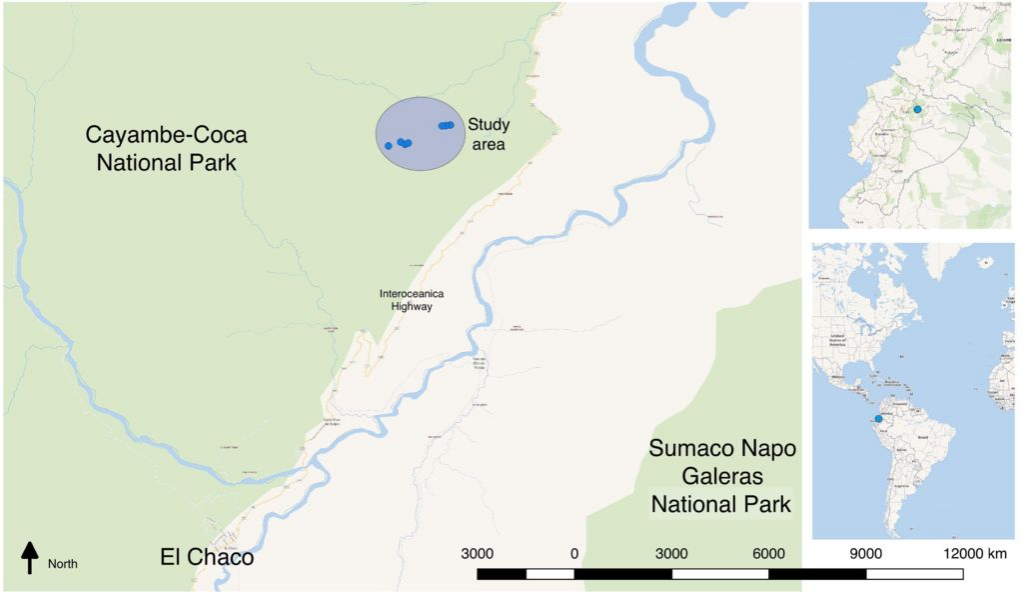
New altitudinal record in Ecuador for *Tayassu pecari*. The study area is outlined in the large light blue circle, with elevations between 1,800 and 2,220 metres altitude. Camera trap locations where peccaries were photographed are denoted with small blue circles.

The study was conducted in a subtropical forest in the north-western Ecuadorian Amazon, close to the city of El Chaco, in Napo Province (Fig. 1), containing an evergreen montane and sub-montane forest ecosystem of the north-eastern Andes in Ecuador (Ministerio del Ambiente del Ecuador 2013). The elevation profile of the surveyed area varied between 1,800 and 2,200 metres above sea level. The site was close to agricultural and cattle areas, but within Cayambe-Coca National Park. The area is inhabited by indigenous populations and colonos, who have several land use practices such as agriculture, cattle and timber extraction. This area is also used for tourism, subsistence and illegal commercial hunting of wildlife (MGP 2011).

Camera traps (Bushnell E3 Trophycam; detection area of approximately 18 metres), were placed along 1 km long transects, in two sites along the study area, within forested areas close to agriculture and cattle pastures, covering a survey area of approximately 1.5 km^2^. At each site, 8 cameras were placed approximately 0.75–1.0 m off the ground, around areas that showed evidence of animal activity, such as tracks and natural paths.

At each site, cameras were continuously active for 2 months, for a total survey time of 127 days (November 2018-March 2019), resulting in a total of 1,016 traps/night. Date and time were automatically stamped on each photograph.

All photographic records were labelled with location, camera, date, time and species. In order to carry out further analyses, we consider consecutive photographs of the same species at a given location to be independent for a species if they were taken at least 30 mins apart (see Blake et al. 2011). To examine daily activity patterns, each record was classified by hour, starting at midnight. Species relative abundance was estimated by multiplying the number of independent records by 100 traps/night. This index is based upon a positive relationship between relative abundance and detection probability, which is expected in camera trap surveys (Nichols and Conroy 1996, Carbone et al. 2001, Hadly and Maurer 2001, Díaz Pulido and Payán Garrido 2012).

## Results

**Figure 2.**
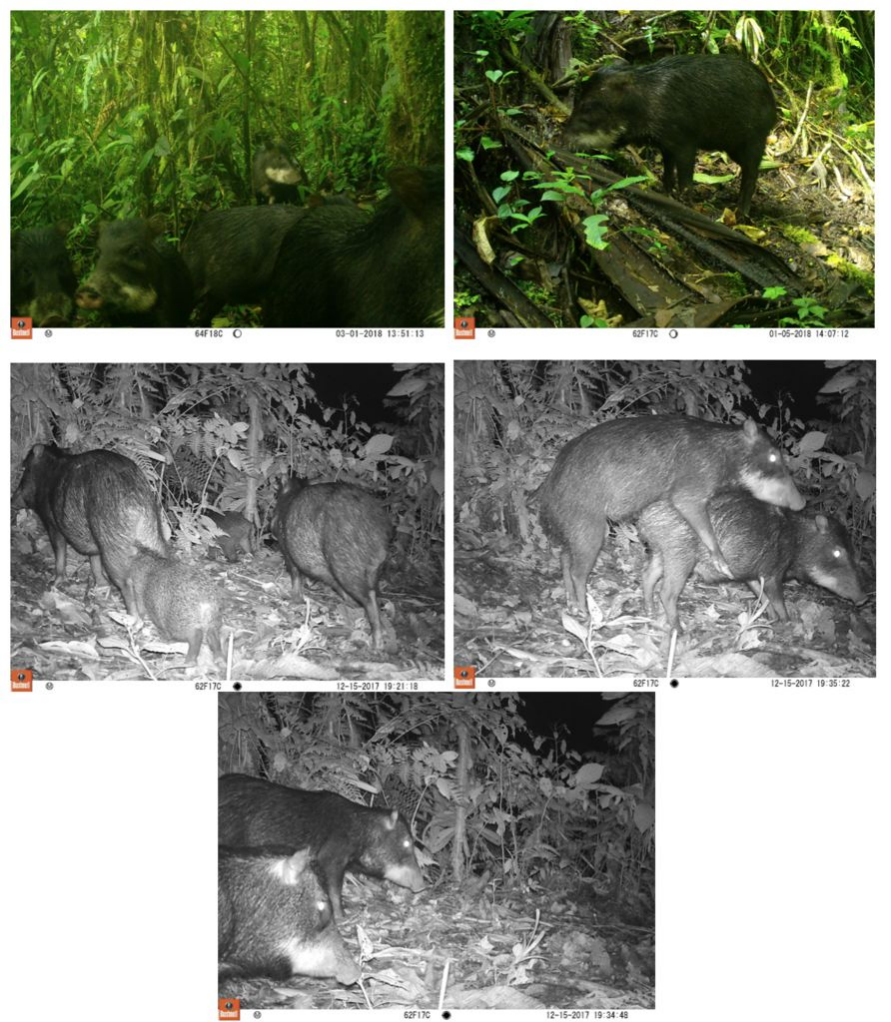
Photographs of white-lipped peccaries (*Tayassu pecari)* taken by camera traps in the study site.

During the study period, we obtained 25 records of white-lipped peccaries (Table 1). In these photographs, we can clearly identify adults, juveniles and newborns during their foraging travels (Fig. 2). These records represent an elevational range expansion for this species in Ecuador, which until now was only known to inhabit tropical and subtropical humid and dry forests, between 0 and 1,600 metres above sea level, according to Tirira (2017). Herd sizes in our study area were much smaller (around 12 individuals) than previously recorded in Ecuador (50 - 300 individuals, Tirira 2017). The estimated relative abundance for white-lipped peccaries in our study area was 2.65 ind/km^2^.

**Figure 3.**
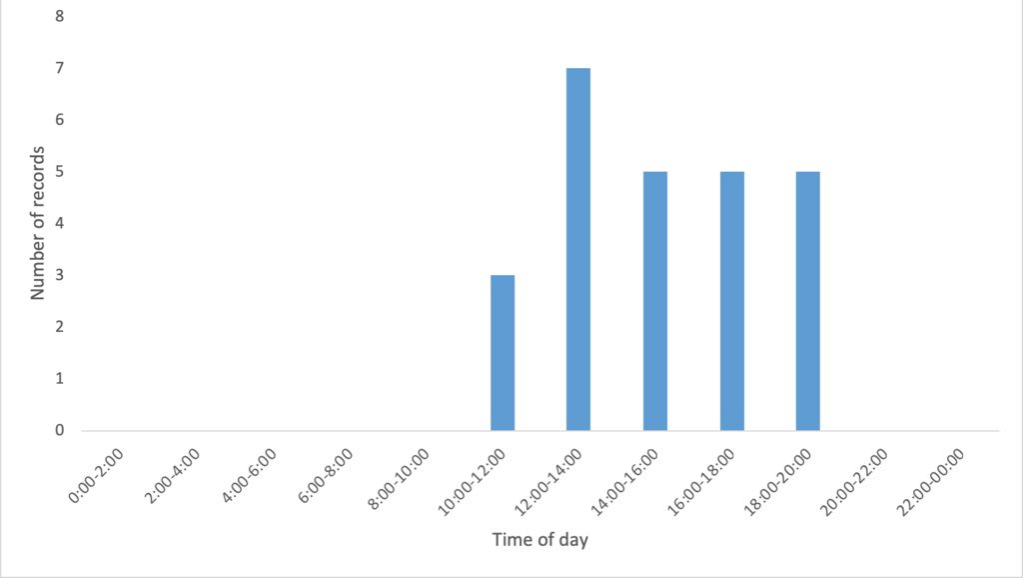
Hourly variation in foraging activity for *Tayassu pecari* recorded in camera traps at the study area.

White-lipped peccaries in our study area were most active in the afternoon: camera traps registered activity for this species between 11:00 am and 7:00 pm, with most of the registries being in the afternoon (Fig. 3). In the photographs of 15 December 2017, we could observe courtship behaviour between a pair of white lipped peccaries, which leads us to infer they were in their reproductive season.

## Discussion

The present study represents a new elevational record for white-lipped peccaries (*Tayassu pecari*) in Ecuador, having recorded the species using camera traps at an elevation of 2,060 m. In addition, we found that these high-altitude peccaries live in herds that are much smaller than those observed elsewhere at lower elevations. Elevation plays a key role in determining group size for vertebrates through a combination of factors such as resource availability, productivity, climatic stress, predation risk and competitive interactions (Chapman and Chapman 2000, Reyna-Hurtado et al. 2012, Paredes et al. 2017). For instance, Dar et al. (2012) found a decrease in density with altitude for ungulate species, which was related to seasonal distribution of water sources, while it has also been reported that small mammal densities tended to decrease with increased elevation (Smith and Merrick 2001, Lomolino 2001). Furthermore, it is important to consider that group-living species, such has white-lipped peccaries, need to acquire sufficient food to meet energetic and nutritional requirements while offsetting the cost of intraspecific feeding competition, thus a positive relationship between resource availability and group size is expected (Reyna-Hurtado et al. 2012, Reyna‐Hurtado et al. 2016, Meyer et al. 2019). This may explain the small herd densities reported at higher elevation sites as those reported in this study.

In Ecuador, white-lipped peccaries (*Tayassu pecari*) have previously been reported occurring up to 1,600 metres above sea level (Tirira 2017), with indirect reports (tracks) for the species in Sardinayacu, Morona Santiago, at an elevation of 1,800 metres (Brito and Ojala-Barbour 2016). According to information from IUCN’s Red List (Keuroghlian et al. 2013), the species usually only ranges up to 1,900 metres, thus our findings might represent a new elevational record for the species in South America.

Interestingly, herds recorded in the study area were small (12 individuals), compared to other records in lowland Amazonia (up to 500 m above sea level), which mostly report herd sizes up to 300 individuals (Sowls 1997, Fragoso 2004, Taber et al. 2008, Tirira 2017). Our new finding might be related to lower resource availability in high elevation areas due to lower productivity compared to lowland areas, resulting in a reduction in population and herd size (Van de Weg et al. 2014). Additionally, the site’s relative closeness to human settlements can be playing a role, which is known to influence white-lipped peccary population size across its distributional range (Reyna‐Hurtado et al. 2016). Another hypothesis that should be tested is whether white-lipped peccary herds divide either spatially or temporally into subgroups or foraging groups as a function of limited resource availability, as reported in some populations in other areas of its range (Carrillo et al. 2002, Keuroghlian et al. 2004). We could not find any other reports about herd sizes of white-lipped peccaries at high altitudes in Ecuador or any other countries in the distributional range.

Our new data on reduced herd size calls into question the genetic viability of these populations, which could face problems such as low genetic diversity, inbreeding and reduced gene flow, which may affect population persistence (Doyle et al. 2015, Willoughby et al. 2015, Li et al. 2016). Even with the data presented herein, some questions remain regarding the threats these populations are facing, such as: how is population genetic structure being maintained for such small populations and are there reproductive problems or diseases in the populations, as have been reported elsewhere in their distribution (Fragoso 2004; de Freitas et al. 2010, Altrichter et al. 2012)? Further work will be necessary to adequately answer these questions.

Finally, our investigation presents diurnal and evening records of activity patterns, that align with previously reported records for the population in Yasuni National Park (Blake et al. 2012) while other investigations have found nocturnal and crepuscular periods of activity in white-lipped peccaries (Fragoso 2004, Taber et al. 2008). These differences in activity patterns between the current and past studies could be related to the environmental pressures and anthropogenic influences (Reyna‐Hurtado et al. 2016) at play in our study area, such as hunting pressure, predator behaviour and resource availability during the study period.

Despite their ecological importance, there have been few studies regarding the population status, distribution and behaviour of white-lipped peccaries in Ecuador; this information is crucial in order to establish successful conservation plans. This report presents important data regarding this species’ current distribution, herd size and activity patterns, in an area that poses significant human-wildlife challenges which will affect peccary populations in the short term.

## Figures and Tables

**Table 1.** GPS coordinates for cameras traps where white-lipped peccaries (*Tayassu pecari)* were registered during the study.

**Cameras trap**	**Coordinates (UTM)**	**Altitude (metres)**
Camera 1 – 2nd site	18M 191486 9972989	2,060
Camera 2 – 1st site	18M 191815 9973094	2,019
Camera 3 – 1st site	18M 191931 9973030	1,984
Camera 4 – 1st site	18M 192019 9973064	1,996
Camera 6 – 1st site	18M 193153 9973547	1,845
Camera 7 – 1st site	18M 193036 9973532	1,852
Camera 8 – 1st site	18M 192028 9973523	1,873

## Hosting institution

Universidad Central del Ecuador - Facultad de Ciencias Biológicas

## References

[B4979937] Altrichter Mariana, Taber Andrew, Beck Harald, Reyna-Hurtado Rafael, Lizarraga Leonidas, Keuroghlian Alexine, Sanderson E. W. (2012). Range-wide declines of a key Neotropical ecosystem architect, the near threatened white-lipped peccary *Tayassu
pecari*. Oryx.

[B4979950] Aranda Marcelo (1994). Importancia de los pecaries (*Tayassu* spp.) En la alimentación del jaguar (*Panthera
onca*). Acta Zoológica Mexicana (nueva serie).

[B4979960] Beck Harald, Thebpanya Paporn, Filiaggi Melissa (2010). Do Neotropical peccary species (Tayassuidae) function as ecosystem engineers for anurans?. Journal of Tropical Ecology.

[B4979970] Blake J. G., Mosquera Diego, Guerra Jaime, Loiselle B. A., Romo David, Swing Kelly (2011). Mineral licks as diversity hotspots in lowland forest of eastern Ecuador. Diversity.

[B4979982] Blake J. G, Mosquera Diego, Loiselle B. A, Swing Kelly, Guerra Jaime, Romo David (2012). Temporal activity patterns of terrestrial mammals in lowland rainforest of eastern Ecuador. Ecotropica.

[B4979994] Briceño-Méndez Marcos, Naranjo E. J, Altrichter Mariana, Mandujano Salvador (2017). Availability of two species of fruits and their influence on the social structure of *Tayassu
pecari* and *Dicotyles
tajacu*. Therya.

[B4980004] Brito Jorge, Ojala-Barbour Reed (2016). Mamíferos no voladores del Parque Nacional Sangay, Ecuador. Papéis Avulsos de Zoologia.

[B4980014] Carbone Chris, Christie S, Conforti K, Coulson T, Franklin N, Ginsberg J. R., Griffiths M, Holden J, Kawanishi K, Kinnaird M (2001). The use of photographic rates to estimate densities of tigers and other cryptic mammals. Animal Conservation.

[B4980030] Carrillo Eduardo, Saenz J. C., Fuller T. K. (2002). Movements and activities of white-lipped peccaries in Corcovado National Park, Costa Rica. Biological Conservation.

[B5073095] Chapman C. A., Chapman L. J. (2000). Constraints on group size in red colobus and red-tailed guenons: examining the generality of the ecological constraints model. *International Journal of Primatology*.

[B5073041] Cullen Laury, Bodmer Richard E., Pádua Claudio Valladares (2000). Effects of hunting in habitat fragments of the Atlantic forests, Brazil. Biological Conservation.

[B5073135] Dar Tanweer A., Habib Bilal, Khan Jamal A. (2012). Group size, habitat use and overlap analysis of four sympatric ungulate species in Shivalik Ecosystem, Uttarakhand, India. Mammalia.

[B5073051] de Azevedo Fernando Cesar Cascelli, Conforti Valéria Amorim (2008). Decline of peccaries in a protected subtropical forest of Brazil: toward conservation issues. mammalia.

[B5073071] de Freitas Tatiana P. Tavares, Keuroghlian Alexine, Eaton Donald P., de Freitas Emanuel Barbosa, Figueiredo Aline, Nakazato Luciano, de Oliveira Jacqueline M., Miranda Flávia, S. Paes Rita Cassia, Carneiro Monteiro Leticia A. R., B. Lima José Vergílio, C. Neto Aparecida A. da, Dutra Valéria, de Freitas Julio Cesar (2010). Prevalence of Leptospira interrogans antibodies in free-ranging Tayassu
pecari of the Southern Pantanal, Brazil, an ecosystem where wildlife and cattle interact. Tropical Animal Health and Production.

[B4980040] Desbiez Arnaud Léonard Jean, Bodmer Richard Ernest, Tomas Walfrido Moraes (2010). Mammalian densities in a Neotropical wetland subject to extreme climatic events. Biotropica.

[B4980050] Díaz Pulido Angélica, Payán Garrido Esteban (2012). Manual de fototrampeo: una herramienta de investigación para la conservación de la biodiversidad en Colombia.

[B4980059] Doyle J. M., Hacking C. C., Willoughby J. R., Sundaram M., DeWoody J. A. (2015). Mammalian genetic diversity as a function of habitat, body size, trophic class, and conservation status. Journal of Mammalogy.

[B4982592] Fragoso J. M. V, Silvius K. M., Bodmer R. E., Fragoso J. M. V. (2004). A long-term study of white-lipped peccary (*Tayassu
pecari*) population fluctuations in northern Amazonia—anthropogenic versus “natural” causes. People in Nature: Wildlife Conservation in South and Central America.

[B4980080] Galetti Mauro, Camargo Hiléia, Siqueira Tadeu, Keuroghlian Alexine, Donatti C. I., Jorge M. L. S. P., Pedrosa Felipe, Kanda C. Z., Ribeiro M. C. (2015). Diet overlap and foraging activity between feral pigs and native peccaries in the Pantanal. PLOS One.

[B4980095] Garla Ricardo C, Setz Eleonore ZF, Gobbi Nivar (2001). Jaguar (*Panthera
onca*) food habits in Atlantic Rain Forest of Southeastern Brazil. Biotropica.

[B4980105] Hadly Elizabeth A, Maurer Brian A (2001). Spatial and temporal patterns of species diversity in montane mammal communities of western North America. Evolutionary Ecology Research.

[B4980125] Keuroghlian Alexine, Eaton Donald P, Longland William S (2004). Area use by white-lipped and collared peccaries (*Tayassu
pecari* and *Tayassu
tajacu*) in a tropical forest fragment. Biological Conservation.

[B4980115] Keuroghlian A., Eaton Donald P (2009). Removal of palm fruits and ecosystem engineering in palm stands by white-lipped peccaries (*Tayassu
pecari*) and other frugivores in an isolated Atlantic Forest fragment. Biodiversity & Conservation.

[B4980364] Keuroghlian A, Reyna-Hurtado R, Altrichter M, Beck H, Taber A, Fragoso JMV (2013). *Tayassu
pecari*. The IUCN Red List of Threatened Species. International Union for Conservation of Nature.

[B4980135] Kiltie R. A., Terborgh John (1983). Observations on the behavior of rain forest peccaries in Perú: Why do white‐lipped peccaries form herds?. Zeitschrift für Tierpsychologie.

[B4980145] Li Haipeng, Xiang-Yu Jinggong, Dai Guangyi, Gu Zhili, Ming Chen, Yang Zongfeng, Ryder O. A, Li W. - H., Fu Y - X, Zhang Y - P (2016). Large numbers of vertebrates began rapid population decline in the late 19th century. Proceedings of the National Academy of Sciences.

[B5073165] Lomolino Mark V. (2001). Elevation gradients of species-density: historical and prospective views. Global Ecology and Biogeography.

[B4980171] Mayer J. J., Wetzel R. M (1987). *Tayassu
pecari*. Mammalian Species.

[B5073175] Meyer Ninon F. V., Moreno Ricardo, Martínez-Morales Miguel Angel, Reyna-Hurtado Rafael (2019). Spatial Ecology of a Large and Endangered Tropical Mammal: The White-Lipped Peccary in Darién, Panama. Movement Ecology of Neotropical Forest Mammals.

[B4980376] MGP (2011). Plan de Desarrollo y Ordenamiento Territorial - Cantón Gonzalo Pizarro.

[B4980161] Ecuador Ministerio del Ambiente del (2013). Sistema de Clasificacion de Ecosistemas del Ecuador Continental.. Quito.

[B4980181] Moreira-Ramírez José Fernando, López Jorge Erwin, García-Anleu Rony, Córdova Francisco, Dubón Tomás (2015). Tamaño, composición y patrones diarios de actividad de grupos de pecarí de labios blancos (*Tayassu
pecari*) en el Parque Nacional Mirador-Río Azul, Guatemala. Therya.

[B4980192] Moreno R S, Kays R W, Samudio Rafael (2006). Competitive release in diets of ocelot (*Leopardus
pardalis*) and puma (*Puma
concolor*) after jaguar (*Panthera
onca*) decline. Journal of Mammalogy.

[B4980202] Nichols J D, Conroy M J (1996). Techniques for Estimating Abundance and Species Richness: Estimation of Species Richness. Smithsonian Institution Press.

[B5073125] Paredes Omar Stalin, Norris Darren, de Oliveira Tadeu Gomes, Michalski Fernanda (2017). Water availability not fruitfall modulates the dry season distribution of frugivorous terrestrial vertebrates in a lowland Amazon forest. PLOS ONE.

[B4980222] Reyna-Hurtado Rafael, Tanner G. W. (2007). Ungulate relative abundance in hunted and non-hunted sites in Calakmul Forest (Southern Mexico). Biodiversity & Conservation.

[B5073061] Reyna-Hurtado Rafael (2009). Conservation Status of the White-Lipped Peccary (Tayassu Pecari) Outside the Calakmul Biosphere Reserve in Campeche, Mexico: A Synthesis. Tropical Conservation Science.

[B4980212] Reyna-Hurtado Rafael, Rojas-Flores Edith, Tanner George W (2009). Home range and habitat preferences of white-lipped peccaries (*Tayassu
pecari*) in Calakmul, Campeche, Mexico. Journal of Mammalogy.

[B5073115] Reyna-Hurtado Rafael, Chapman Colin A., Calme Sophie, Pedersen Eric J. (2012). Searching in heterogeneous and limiting environments: foraging strategies of white-lipped peccaries (Tayassu
pecari). Journal of Mammalogy.

[B4980232] Reyna‐Hurtado Rafael, Beck Harald, Altrichter Mariana, Chapman C. A., Bonnell T. R., Keuroghlian Alexine, Desbiez A. L., Moreira‐Ramírez J. F., O'Farrill G., Fragoso J. (2016). What ecological and anthropogenic factors affect group size in white‐lipped peccaries (*Tayassu
pecari*)?. Biotropica.

[B4980248] Ringler Max, Hödl Walter, Ringler Eva (2015). Populations, pools, and peccaries: simulating the impact of ecosystem engineers on rainforest frogs. Behavioral Ecology.

[B5073145] Smith Rosemary J., Merrick Melissa J. (2001). Resource availability and population dynamics of Nicrophorus investigator, an obligate carrion breeder. Ecological Entomology.

[B4980258] Sowls L. K (1997). Javelinas and other peccaries: their biology, management, and use.

[B4980267] Taber A, Chalukian S. C., Altrichter M, Minkowski K, Lizarraga L, Sanderson E, Rumiz D, Ventincinque E, Moraes E. A., Angelo C (2008). El destino de los arquitectos de los bosques neotropicales: evaluación de la distribución y el estado de conservación de los pecaríes labiados y los tapires de tierras bajas. Wildlife Conservation Society, Wildlife Trust.

[B4980283] Tirira Diego (2001). Libro Rojo de los Mamíferos del Ecuador.

[B4980292] Tirira Diego (2017). Guía de campo de los mamíferos del Ecuador. Incluye las Islas Galápagos y la Zona Antártica Ecuatoriana. Editorial Murciélago Blanco.

[B4980312] Van de Weg M. J., Meir Patrick, Williams Mat, Girardin Cécile, Malhi Yadvinder, Silva-Espejo Javier, Grace John (2014). Gross primary productivity of a high elevation tropical montane cloud forest. Ecosystems.

[B4980325] Willoughby J. R, Sundaram Mekala, Wijayawardena B. K., Kimble S. J. A., Ji Yanzhu, Fernandez N. B., Antonides J. D, Lamb M. C, Marra N. J., DeWoody J. A. (2015). The reduction of genetic diversity in threatened vertebrates and new recommendations regarding IUCN conservation rankings. Biological Conservation.

